# STK4 is a prognostic biomarker correlated with immune infiltrates in clear cell renal cell carcinoma

**DOI:** 10.18632/aging.205127

**Published:** 2023-10-20

**Authors:** Zi-Yuan Bai, Lu-Shan Peng, Run-Qi Li, Xianchu Peng, Zhe Yang

**Affiliations:** 1 Department of Pathology, Xiangya Hospital, Central South University, Changsha 410008, Hunan, China; 2 Department of Pathology, First Affiliated Hospital of Kunming Medical University, Kunming 650032, Yunnan, China; 3 School of Basic Medicine, Central South University, Changsha 410031, Hunan, China; 4 National Clinical Research Center for Geriatric Disorders, Xiangya Hospital, Central South University, Changsha 410008, Hunan, China; 5 Key Laboratory of Hunan Province in Neurodegenerative Disorders, Xiangya Hospital, Central South University, Changsha 410008, Hunan, China

**Keywords:** STK4, clear cell renal cell carcinoma, immune infiltration, biomarker, prognosis

## Abstract

Background: Mammalian STE20-like kinase 1 (MST1/STK4/KRS2) is a highly conserved serine/threonine kinase and a central member of the Hippo signaling pathway. STK4 has been reported to play important roles in various tumors, but a systematic and comprehensive study of its function in clear cell renal cell carcinoma (ccRCC) has not been conducted.

Methods: In this study, we used immunohistochemistry (IHC), western blot (WB), quantitative real-time PCR (qPCR) experiments, and bioinformatics analysis to comprehensively analyze the expression, prognostic value, and immune infiltration of STK4 in ccRCC.

Results: Analysis of the TCGA database showed that the expression level of the STK4 gene in ccRCC patients depended on tumor stage, grade, and distant lymphatic metastasis. This was further confirmed by the results of IHC, WB, and qPCR. In addition, we used the receiver operating characteristic curve (ROC curve) to elucidate the diagnostic value of STK4 in ccRCC patients. According to the findings of the TIMER database, the high expression of STK4 is significantly associated with the survival of kidney cancer (including ccRCC) patients (*p* < 0.001), suggesting that STK4 is a reliable prognostic predictor. We then used gene set enrichment analysis (GSEA) to explore the mechanisms behind STK4 function in ccRCC. We found that STK4 may play a role in immune regulation interactions. Subsequently, we performed immune infiltration analysis of STK4. The results showed that STK4 may regulate the development of ccRCC by affecting the immune infiltration of NK and pDC cells.

Conclusions: STK4 may be a prognostic marker for ccRCC and may help identify new strategies for treating ccRCC patients.

## INTRODUCTION

Clear cell renal cell carcinoma (ccRCC) is the most common type of renal tumors, accounting for approximately 85–90% of cases, and is characterized by high mortality rates and poor treatment response [1, 2]. Despite various treatment options, ccRCC often exhibits resistance to radiation, cytotoxicity, and hormonal therapies, leading to a mortality rate of 60% within the first 2–3 years, with 30% of patients diagnosed with metastasis, the 5-year survival rate for these patients with metastatic ccRCC was only 8% [3, 4]. Therefore, there is an urgent need to identify new biomarkers for the early diagnosis of ccRCC.

Mammalian STE20-like kinase 1 (MST1/STK4/KRS2) is a highly conservative serine/threonine kinase, as well as a core member of the Hippo signaling pathway, which plays a role in regulating cell cycle, promoting apoptosis and inhibiting tumor growth [5, 6]. It has been reported that STK4 plays important roles in tumorigenesis in multiple cancers including prostate cancer [7], breast cancer [8], and hepatocellular carcinoma [9]. In this study, we aimed to investigate the potential role of STK4 as a new biomarker for the early detection of ccRCC. While STK4 has been reported to have tumor suppressive activities in various cancers, its role in ccRCC remains understudied. Surprisingly, we found that STK4 may have tumor-promoting effects in our study. It is speculated that the different roles played by STK4 in various tumors may be associated with tumor heterogeneity. According to previous research, this function may be related to STK4’s regulation of YAP1. Rybarczyk et al. reported that YAP1 acts as an oncogene in ccRCC cells, promoting cell proliferation and survival [10]. Over, another study found that cytoplasmic YAP1 in ccRCC patients was associated with poor prognosis and higher risk of death [11]. Therefore, we hypothesize that the upregulation of STK4 limits YAP1 translocation to the nucleus, causing YAP1 to remain in the cytoplasm and further promoting ccRCC proliferation and progression.

In this study, we explored the potential role of STK4 in ccRCC tumorigenesis and emphasized the relationship between STK4 expression and clinical pathological characteristics of patients. Based on our comprehensive analysis, we believe that STK4 is a promising diagnostic and prognostic biomarker for ccRCC.

## MATERIALS AND METHODS

### RNA-sequencing data and bioinformatics analysis

We used the TCGA database (https://portal.gdc.cancer.gov/) to analyze the transcriptional levels of STK4. The downloaded data we analyzed included 539 ccRCC cases (72 of which had paired adjacent tissues) expressed in level 3 HTSeq-fragments per kilobase per million (FPKM) format, and 531 ccRCC cases (100 of which had paired adjacent tissues) expressed in transcripts per million (TPM) formats. All operations in this study complied with the Helsinki Declaration (revised in 2013).

### Gene set enrichment analysis (GSEA)

The GSEA and MSigDB v6.2 (Molecular Signatures Database) were downloaded from the GSEA website (http://software.broadinstitute.org/gsea/index.jsp) for the purpose of GSEA analysis between the high-STK4 and low-STK4 groups [12]. A false discovery rate (FDR) *q*-value < 0.25 and adjusted *p*-value < 0.05 were considered significant enrichments.

### Immune infiltration analysis by ssGSEA

The immune infiltration analysis of ccRCC was performed by single sample GSEA (ssGSEA) method from R package GSVA (version 1.34.0) (http://www.bioconductor.org/packages/release/bioc/html/GSVA.html), and the infiltration of 24 immune cell types from gene expression profile in the literature was quantified [13, 14]. The Spearman and Wilcoxon rank sum tests were used to determine the *p* values to discover the correlation between STK4 and the infiltration levels of 24 immune cells.

### Immunohistochemistry

We obtained 10 specimens of clear cell renal cell carcinoma from the Pathology Department of Xiangya Hospital, Central South University, and another 10 specimens from the Pathology Department of the First Affiliated Hospital of Kunming Medical University. We used STK4 (Abclonal, Woburn, MA, USA, A8043, 1:200) as primary antibodies, and incubated with it overnight at 4°C. Then, the sections were incubated with the secondary antibodies and DAB regents for staining (Zhong Shan Golden Bridge Biotechnology, Beijing, China).

### Cell culture

The human renal epithelial cell line 293T and human ccRCC cell lines 786-0, Caki, ACHN, and A498 were sourced from the Kunming Institute of Zoology, Chinese Academy of Sciences (Kunming, China) and were cultured in DMEM medium (11875-085, Gibco, Grand Island, NY, USA) containing 10% FBS (16140071, Thermo Fisher Scientific, Waltham, MA, USA) at 37°C in a CO_2_ incubator (5% CO_2_). No contamination of mycoplasma was found in these cell lines.

### Western blotting

Total protein was extracted from cells using RIPA Lysis Buffer (Promega, Madison, WI, USA), separated on a 10% SDS-PAGE gel, and transferred onto a PVDF membrane. The membrane was blocked with 5% skim milk at room temperature for 1.5 hours, incubated with primary antibody (Abclonal, A8043, 1:1000) overnight at 4°C, and then incubated with secondary antibody (1:5000) conjugated with horseradish peroxidase (HRP) at room temperature for 60 minutes. The protein bands were scanned and visualized using a GS700 imaging densitometer (Bio-Rad Laboratories, Hercules, CA, USA), and analyzed using Image Studio software.

### PCR

Total RNA was isolated and real-time RT-PCR amplifications were performed according to previous reports using the following primers: U6: F: 5′-CTCGCTTCGGCAGCACA-3′, R: 5′-AACGCTTCACGAATTTGCGT-3′; STK4: F: 5′-GAGCAGGAGATTGAAGAGAT-3′; 5′-AGAGACGACAGAGCAGAA-3′.

### TIMER

TIMER is a comprehensive database with visualization of tumor-infiltrating immune cells and is applicable to various cancer types [15]. In this study, the TIMER tool was used to explore the expression of the STK4 gene between tumor and normal tissues.

### UALCAN

An effective online cancer data analysis website, mainly based on the relevant cancer data in the TCGA database [16]. This website can identify biomarkers, analyze gene expression profiles, and conduct survival analysis. We used this database to evaluate the mRNA expression of STK4 and conducted expression analysis based on tumor stage, grade, and lymph node metastasis status.

### The Human Protein Atlas

The Human Protein Atlas is a database based on proteomics, transcriptomics, and systems biology data, which can generate maps of tissues, cells, and organs. It includes not only protein expression in tumor tissues but also in normal tissues. Moreover, it provides survival curves of cancer patients. In this study, we utilized this database to explore the correlation between STK4 expression and patient survival. Patients were divided into two groups, “low” (below the cut-off value) and “high” (above the cut-off value), based on the expression level. The current cut-off value is the optimal expression cut-off value, which was chosen based on survival analysis. X-axis shows time for survival (years) and y-axis shows the probability of survival, where 1.0 corresponds to 100 percent.

## RESULTS

### STK4 expression in patients with ccRCC

**Figure 1:**
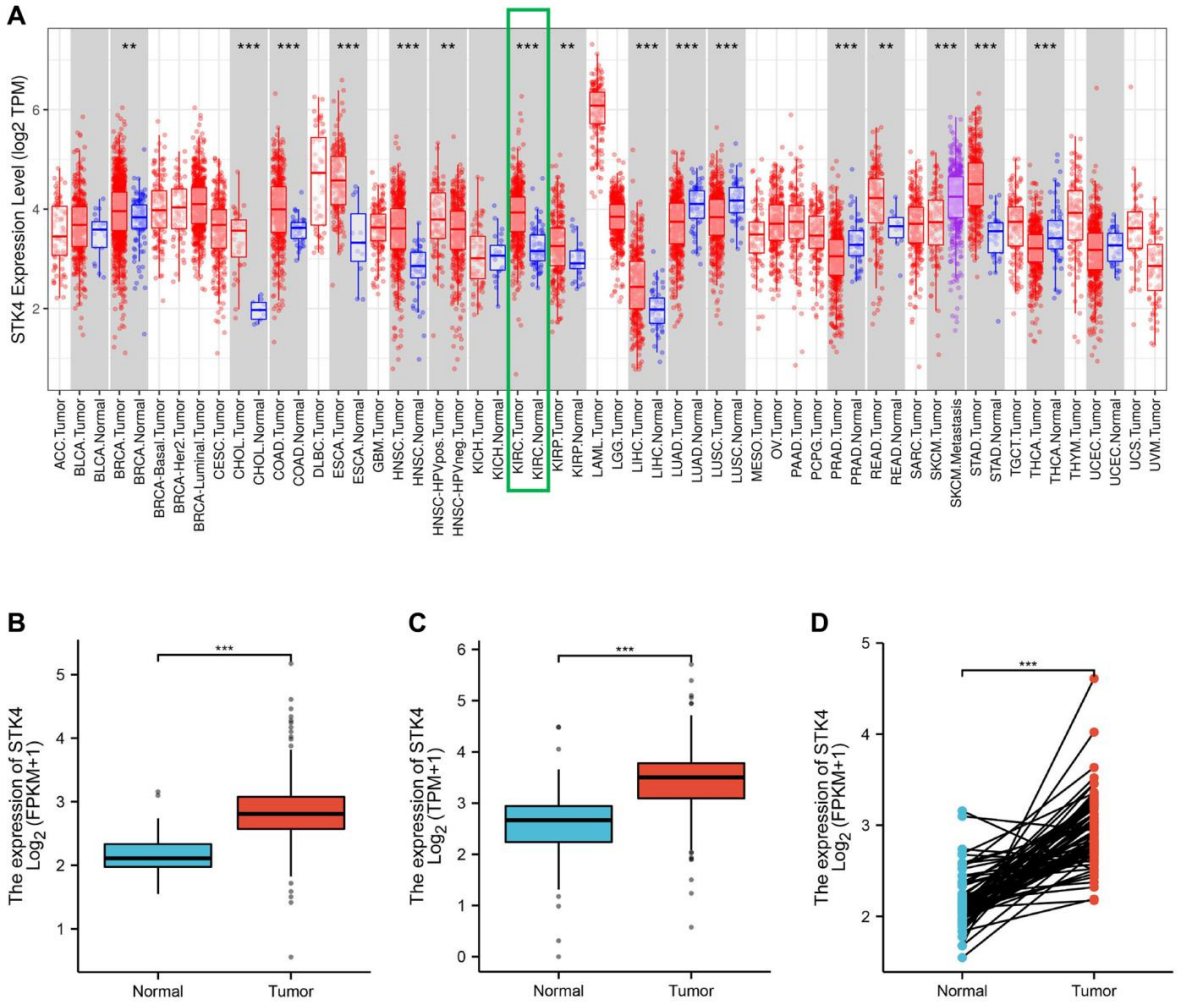
**Differential expression of STK4 between tumors and adjacent normal tissues.** STK4 expression in multiple tumors and normal tissues based on the TIMER database (**A**). STK4 expression in ccRCC tissues and normal tissues by using GTEx database and TCGA database (*p* < 0.001) (**B**, **C**). STK4 expression in ccRCC tissues and adjacent tissues (*p* < 0.001) (**D**).

Initially, we compared the expression levels of STK4 in cancer and normal tissues using the TIMER database. We found that STK4 was highly expressed in various types of cancer, including ccRCC (Figure 1A). Next, we further analyzed the expression levels of STK4 using the TCGA database. The downloaded data included 539 cases of ccRCC (of which 72 had paired adjacent tissue). Compared with adjacent normal tissue, the expression of STK4 in ccRCC was significantly increased (*p* < 0.001, Figure 1B). The analysis of ccRCC and its matched tissue showed similar results (*p* < 0.001, Figure 1C). Additionally, we compared the expression of STK4 in normal samples from the GTEx database with that in ccRCC samples from the TCGA database. The results also showed overexpression of STK4 in ccRCC samples (*p* < 0.001, Figure 1D).

### Experiments verification of the expression of STK4 in ccRCC

**Figure 2:**
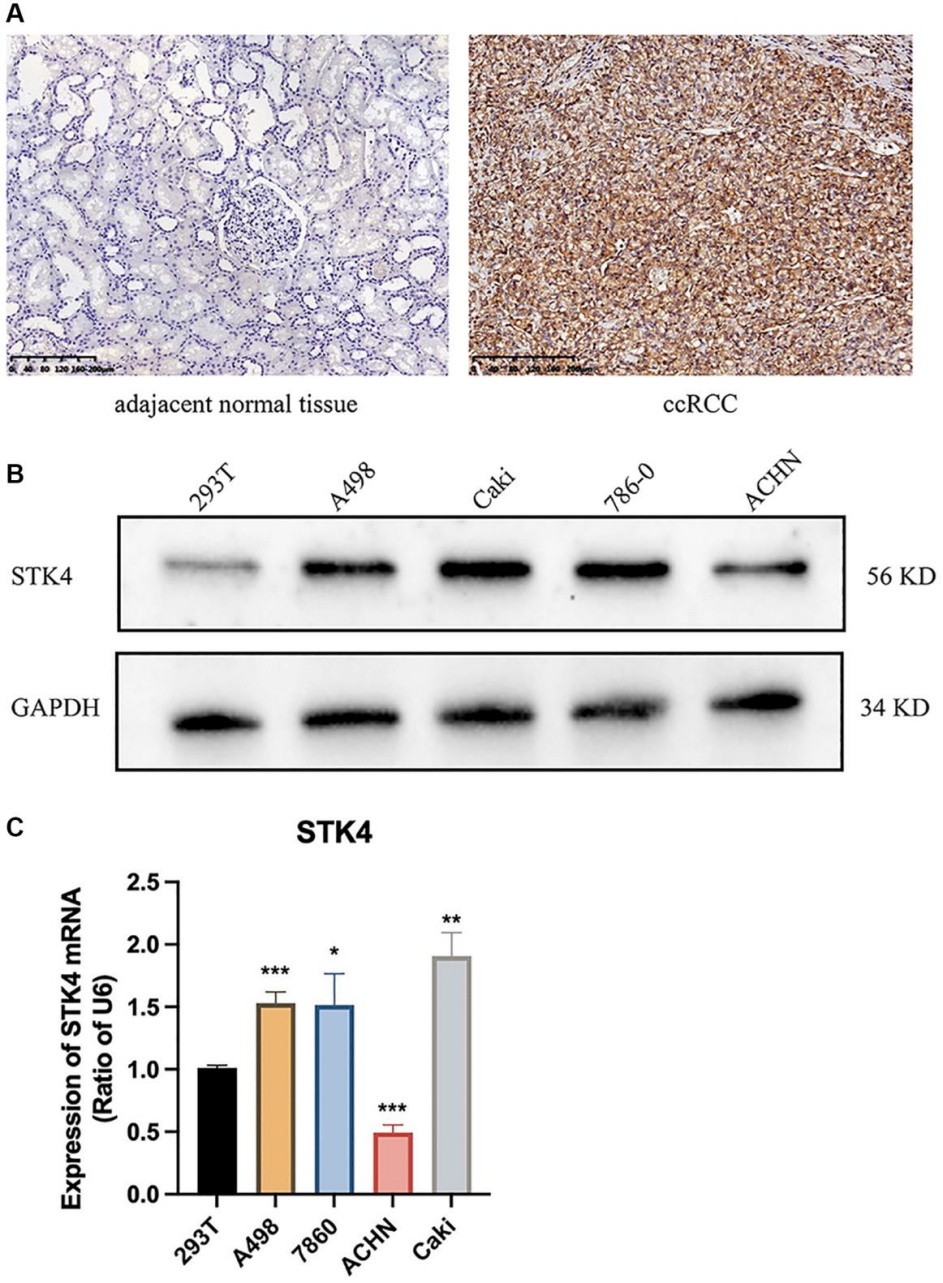
**Experiment results of STK4 expression in ccRCC.** IHC (**A**), WB (**B**), and q-pCR (**C**) results of STK4 expression in ccRCC.

To further validate the expression of STK4 in ccRCC, we conducted immunohistochemistry staining (IHC), western blotting (WB), and quantitative PCR (q-PCR) experiments. The results in Figure 2A and Supplementary Figure 1 showed that the expression of STK4 in ccRCC tissue was significantly increased compared with normal kidney tissue. The WB results showed that the expression of STK in human ccRCC cell lines (ACHN, 786-0, Caki, and A498) was higher than that in human renal epithelial cell lines (293T) (Figure 2B). q-PCR also showed the same results (Figure 2C).

### Clinical-pathological characterizations of STK4 in patients with ccRCC

First, we collected the clinical characteristics of 539 ccRCC patients including TNM stage, pathologic stage, histologic grade, gender, age, and OS (Table 1). This study includes 186 female and 353 male, 49.9% of them are ≤60 (*n* = 269), while 50.1% of them are >60 (*n* = 270). In TNM stage, we found that there are 278 (51.6%) patients in T1, 71 (13.2%) in T2, 179 (33.2%) in T3, 11 (2%) in T4, 241 (93.8%) in N0, 16 (6.2%) in N1, 428 (84.6%) in M0, and 78 (15.4%) in M1. Regarding tumor pathologic stage, stage I was found in 272 (50.7%) patients, stage II was in 59 (11%), Stage III in 123 (22.9%), and stage IV in 82 (15.3%). The histologic grade included 2.6% G1 (*n* = 14), 44.3% G2 (*n* = 235), 39% G3 (*n* = 207), and 14.1% G4 (*n* = 75). Among these 539 patients, 366 (67.9%) were still alive, and 173 (32.1%) were found dead.

**Table 1:** The clinical characteristics of 539 ccRCC patients.

**Characteristic**	**Levels**	**Overall**
*n*		539
T stage, *n* (%)	T1	278 (51.6%)
	T2	71 (13.2%)
	T3	179 (33.2%)
	T4	11 (2%)
N stage, *n* (%)	N0	241 (93.8%)
	N1	16 (6.2%)
M stage, *n* (%)	M0	428 (84.6%)
	M1	78 (15.4%)
Pathologic stage, *n* (%)	Stage I	272 (50.7%)
	Stage II	59 (11%)
	Stage III	123 (22.9%)
	Stage IV	82 (15.3%)
Histologic grade, *n* (%)	G1	14 (2.6%)
	G2	235 (44.3%)
	G3	207 (39%)
	G4	75 (14.1%)
Gender, *n* (%)	Female	186 (34.5%)
	Male	353 (65.5%)
Age, *n* (%)	≤60	269 (49.9%)
	>60	270 (50.1%)
OS event, *n* (%)	Alive	366 (67.9%)
	Dead	173 (32.1%)

**Figure 3:**
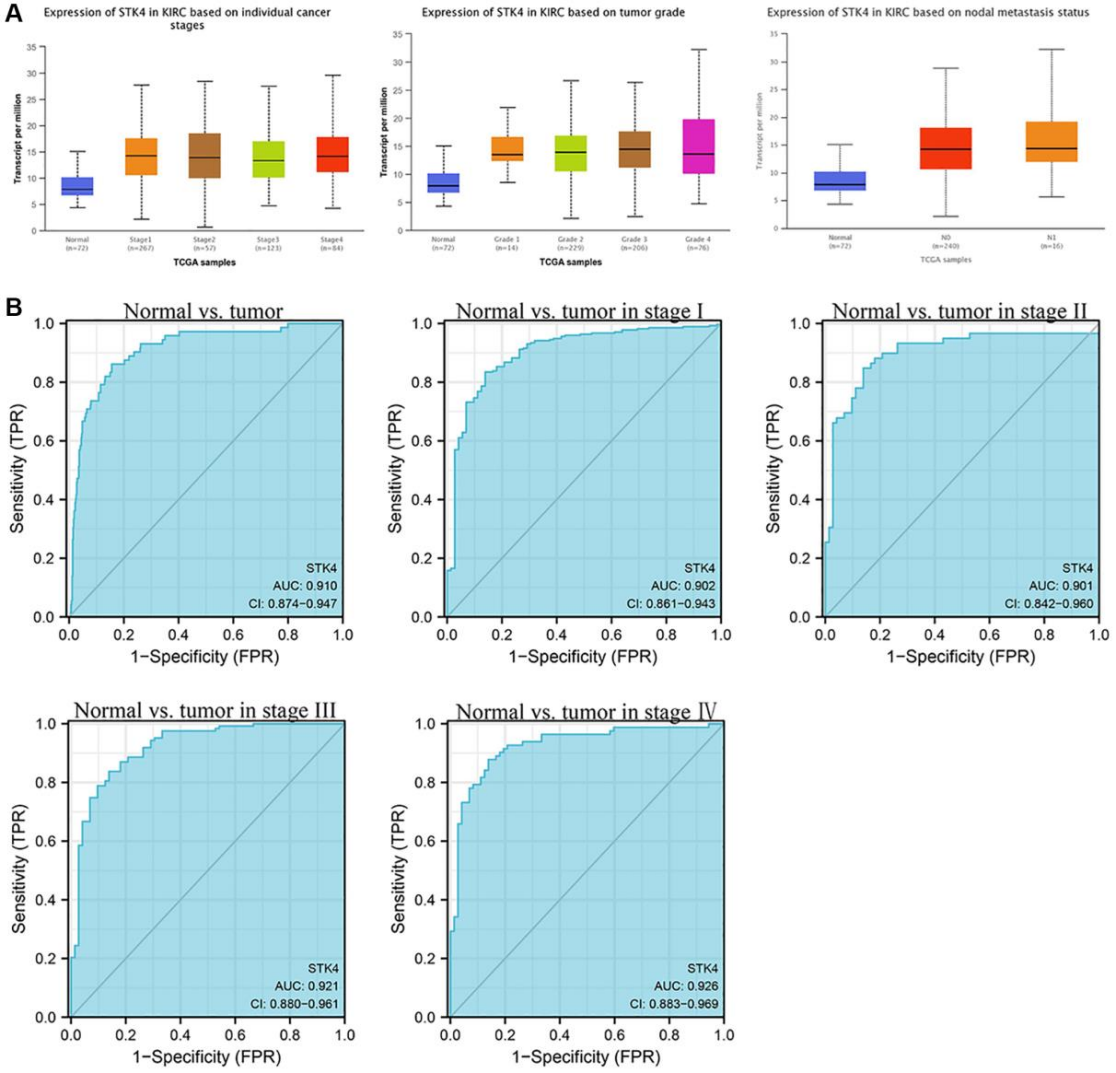
**STK4-associated clinical pathology characteristics of patients with ccRCC (also called kidney renal clear cell carcinoma, KIRC).** (**A**) The expression of STK4 is based on the tumor stage, tumor grade, and nodal metastasis status. (**B**) The ROC curve of STK4 in patients with ccRCC.

Next, to further validate the influence of STK4 on the clinical pathological characteristics of patients with ccRCC. We used the UALCAN database to investigate the relationship between the expression of STK4 and tumor stages, grades, and nodal metastasis status (Figure 3A). The results showed that STK4 expression was significantly upregulated in stage I–IV, grade I–IV, and M0–1.

### The effectiveness of STK4 as a diagnostic biomarker in ccRCC

In order to clarify the diagnostic value of STK4 in patients with ccRCC, we used the receiver operating characteristic (ROC) curve to distinguish ccRCC tissues from adjacent normal tissues (Figure 3B). We found that the area under the curve (AUC) of STK4 was 0.910, indicating that STK4 may be a possible biomarker. Then in tumor stage I-IV, the results showed that their AUC are 0.902, 0.901, 0.921, and 0.926 respectively, with diagnostic values.

### Association between poor prognosis and STK4 in patients with ccRCC

**Figure 4:**
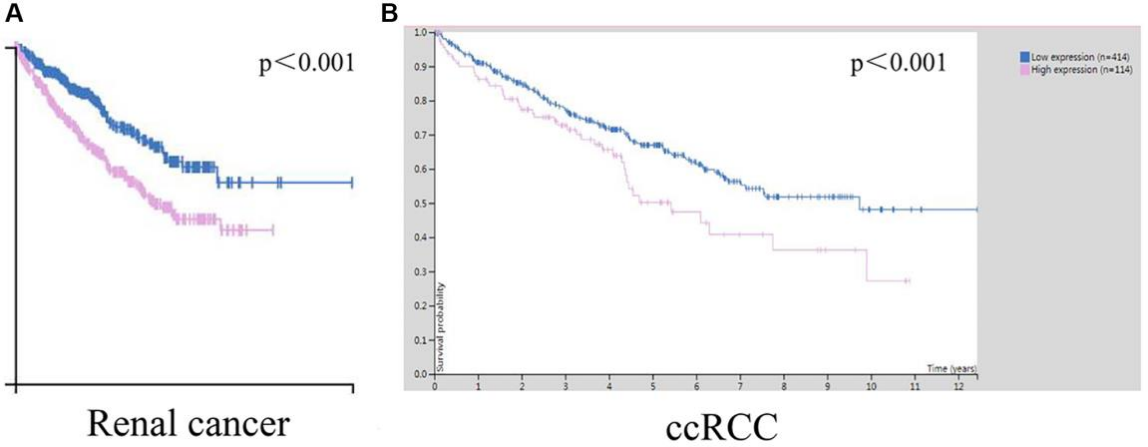
**The prognostic value of STK4 in ccRCC patients.** The relationships between STK4 transcript levels and OS of patients with renal cancer (**A**) and ccRCC (**B**).

To explore the influence of STK4 on the prognosis of ccRCC patients, Kaplan-Meier plots were used to draw the overall survival (OS) curve. We found that the high expression of STK4 has a significant (*p* < 0.001) association with renal cancer (including ccRCC) patients’ survival (Figure 4A, 4B), suggesting that STK4 is a reliable prognostic predictor.

### STK4-related functional enrichment analysis

**Figure 5:**
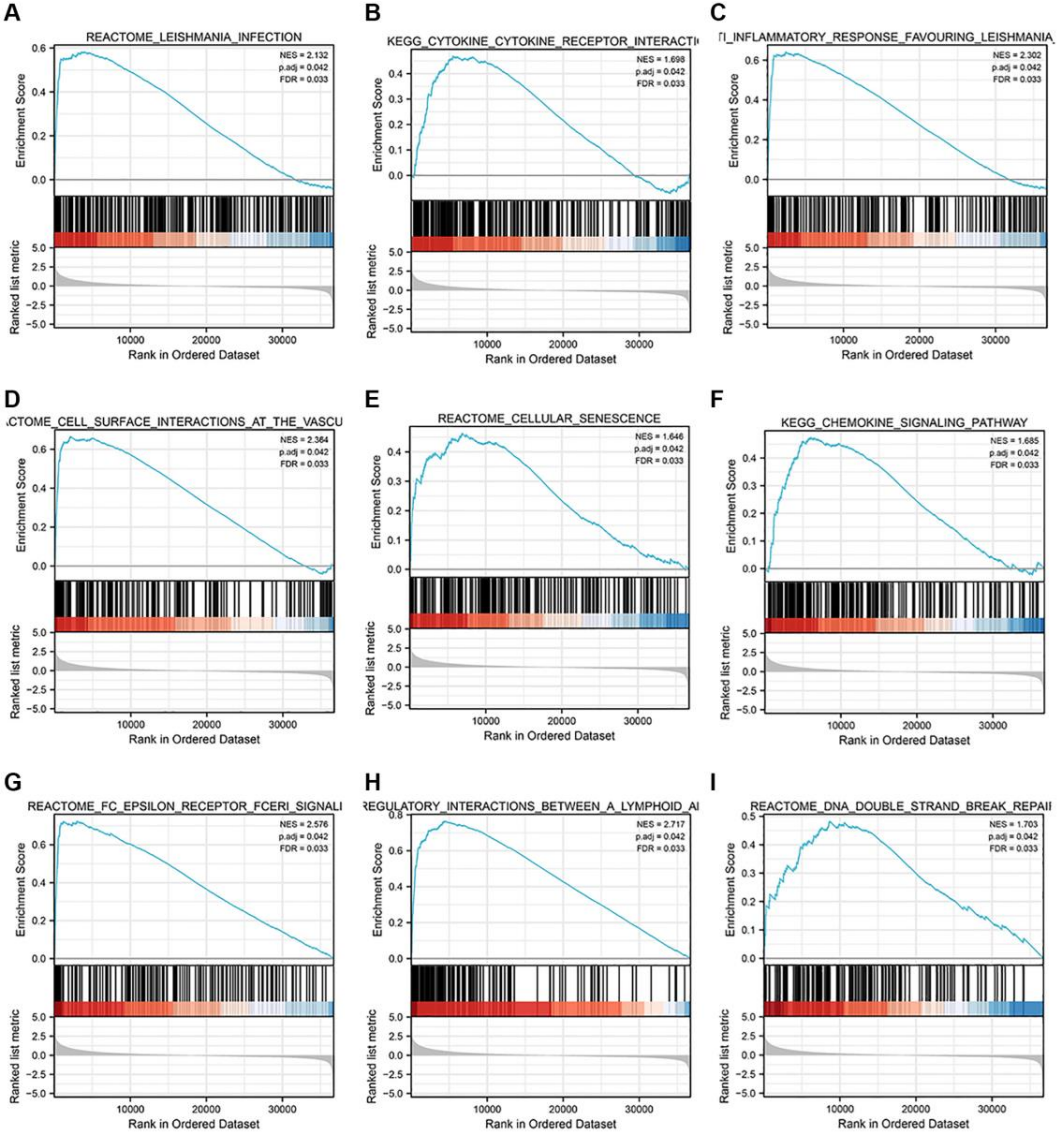
**STK4-related functional enrichment analysis by GSEA.** Top 9 highly enriched STK4-associated signaling pathways in ccRCC (**A**–**I**).

To explore STK4 associated signaling pathway in ccRCC for identifying the mechanism underlying the functions of STK4 in ccRCC, GSEA was performed. FDR < 0.05, NOM *p*-value < 0.05 was thought with significant differences. Results showed differential enrichment in leishmaniasis, cytokine receptor interactions, anti-inflammatory response, vascular endothelial cell surface interactions, cellular aging, chemokine signaling pathways, FC epsilon receptor I signaling pathway, immune regulation interactions in lymphoid and non-lymphoid cells, and DNA double-strand break repair in PRAS40 mRNA expression with positive correlation phenotypes (Table 2, Figure 5).

**Table 2:** STK4-related functional enrichment analysis in ccRCC patients.

**Gene set name**	**NES**	**MOM *p*-val**	**FDR *q*-val**
REACTOME_LEISHMANIA_INFECTION	2.132326419	0.001037344	0.032866978
KEGG_CYTOKINE_CYTOKINE_RECEPTOR_INTERACTION	1.697808178	0.00105042	0.032866978
REACTOME_ANTI_INFLAMMATORY_RESPONSE_FAVOURING_LEISHMANIA_PARASITE_INFECTION	2.301769666	0.001064963	0.032866978
REACTOME_CELL_SURFACE_INTERACTIONS_AT_THE_VASCULAR_WALL	2.364293608	0.001081081	0.032866978
REACTOME_CELLULAR_SENESCENCE	1.64642411	0.001081081	0.032866978
KEGG_CHEMOKINE_SIGNALING_PATHWAY	1.685414905	0.001084599	0.032866978
REACTOME_FC_EPSILON_RECEPTOR_FCERI_SIGNALING	2.575610997	0.001086957	0.032866978
REACTOME_IMMUNOREGULATORY_INTERACTIONS_BETWEEN_A_LYMPHOID_AND_A_NON_LYMPHOID_CELL	2.717181942	0.001086957	0.032866978
REACTOME_DNA_DOUBLE_STRAND_BREAK_REPAIR	1.702866631	0.00110011	0.032866978

### The relationship between STK4 expression and immune infiltration

**Figure 6:**
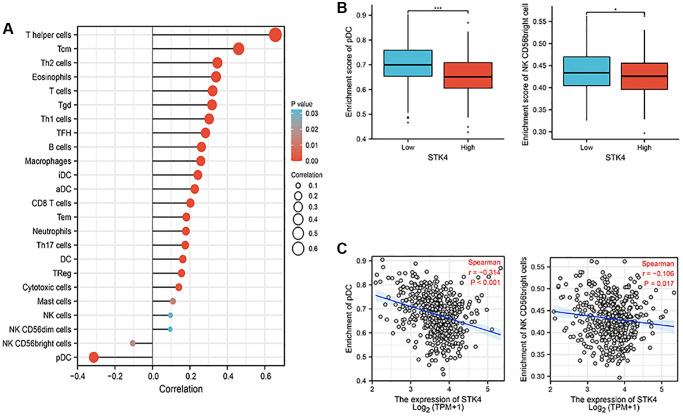
**Association between the STK4 expression and immune infiltration in the tumor microenvironment.** (**A**) The forest plot shows the correlation between STK4 expression and multiple immune cells. The absolute value of Spearman r was indicated by the size of the dots. (**B**) The Wilcoxon rank sum test was used to analyze the difference in pDC and NK cells infiltration levels between STK4 high and low expression groups. (**C**) The Spearman correlation method was used to analyze the correlation between STK4 expression and pDC and NK cells infiltration.

Nowadays, immune cells have been considered a crucial aspect in understanding oncogenesis and cancer progression because of their important role in the tumor microenvironment [17, 18]. Based on the STK4-related functional enrichment analysis above, we also found that STK4 may play roles in immunoregulatory interactions. So, ssGSEA with Spearman R was used to analyze the correlation between the expression of STK4 and immune infiltration. We found that STK4 expression was negatively correlated with plasmacytoid dendritic cells (pDCs) (*p* < 0.001) and natural killer (NK) CD56 bright cells (*p* = 0.017), while STK4 expression was positively correlated with several immune cells including T helper cells, Tcm, Th2 cells and so on (Figure 6). Therefore, we infer that STK4 may regulate immune infiltration and play a role in the development of ccRCC by affecting NK and pDCs cells.

## DISCUSSION

As the most common urinary system tumor, ccRCC accounts for approximately 85–90% of cases and is characterized by a high degree of initial malignancy, insidious onset, rapid disease progression, and susceptibility to resistance to targeted drugs [1, 2]. STK4 is a highly conservative serine/threonine kinase, as well as a core member of the Hippo signaling pathway. Its crucial role in various cancers, including prostate cancer, breast cancer, and hepatocellular carcinoma, has been previously reported [7–9]. In these cancers, STK4 has often been described as a tumor suppressor, inhibiting cancer through the regulation of cell cycle and promotion of apoptosis. However, the investigation of STK4 in ccRCC is currently limited. We found that STK4 may work as a tumor promoter in ccRCC. We speculated that the different roles of STK4 toward tumors are related to the heterogeneity of cancer. And this function may be related to the regulatory function of STK4 on YAP1 [10, 11]. Therefore, we propose that STK4 could serve as a potential biomarker for ccRCC.

In our study, we first conducted bioinformatics analysis using the TCGA database and UALCAN database. The results showed that the expression of STK4 in ccRCC was associated with tumor stage, tumor grade, and lymph node metastasis status. To validate this result, we performed IHC of STK4 on 20 specimens from 2 hospitals. The experimental results were consistent with the database search results, indicating high expression of STK4 in ccRCC tissues. Furthermore, we also conducted WB and q-PCR on human renal epithelial cell line 293T and ccRCC cell lines 786-0, Caki, ACHN, and A498. The WB results were consistent with the bioinformatics and IHC results. In q-PCR, the expression level of STK4 was significantly higher in the 786-0, Caki, and A498 cell lines compared to the 293T cell line. However, STK4 was downregulated in the ACHN cell line. This is because the decrease in mRNA expression can be attributed to factors that stabilize mRNA, such as increasing the length of the polyA tail (by increasing the activity of polyA polymerase) or decreasing mRNA degradation, resulting in an extension of mRNA half-life and an increase in the protein level. In addition, mRNA levels decrease, but proteins undergo modifications to reduce protein degradation (such as the ubiquitin-proteasome system), extending the half-life of the proteins and thus increasing their abundance. This is also a possibility. The ACHN cell line originates from pleural effusion of a 22-year-old Caucasian male with renal cell adenocarcinoma rather than from the primary renal tumor. Therefore, there may exist different transcription modifications and post-transcriptional modifications within ACHN cell line. Then, the ROC curve results suggested the diagnostic value of STK4 in patients with ccRCC. Next, according to the OS curve, we found that the high expression of STK4 has a significant (*p* < 0.001) association with renal cancer (including ccRCC) patients’ survival, which further suggests that STK4 is a reliable prognostic predictor. Through KEGG enrichment analysis by using GSEA to explore the mechanism underlying the functions of STK4 in ccRCC. We found that STK4 may play roles in immunoregulatory interactions (immunoregulatory interactions between a lymphoid and a non-lymphoid cell). Besides, leishmania infection, cytokine receptor interaction, anti-inflammatory response, cell surface interaction at the vascular wall, cellular senescence, chemokine signaling pathway, FC epsilon receptor fceri signaling, and DNA double strand break repair may also be closely related to the progression of ccRCC. Subsequently, STK4 was subjected to immune infiltration analyses. Results suggested that STK4 may play roles in the development of ccRCC by adjusting immune infiltration by influencing NK and pDCs cells. We plan to explore the critical role of immune cell infiltration in antitumor immunity in the future.

## CONCLUSIONS

In conclusion, the molecular profiles of the SKT4 were analyzed by using bioinformatic methods and experiments including q-PCR and IHC together. Combined with experiments, we suggested that the expression of SKT4 may be important for promoting the tumorigenesis of ccRCC. Specifically, the mechanism may be related to the infiltration of immune cells. We speculated that these findings could provide more details for understanding ccRCC, which could help predict its prognosis and identify new treatment strategies.

## Supplementary Materials

Supplementary Figure 1
