# Supplementary material for: STK4 is a prognostic biomarker correlated with immune infiltrates in clear cell renal cell carcinoma
Source: Aging (Albany NY). 2023 Oct 20;15(20):11286–97. doi: 10.18632/aging.205127 (PMC10637789; doi:10.18632/aging.205127)
Supplement: Supplementary Figure 1 [file aging-15-205127-s001.pdf]

SUPPLEMENTARY FIGURE

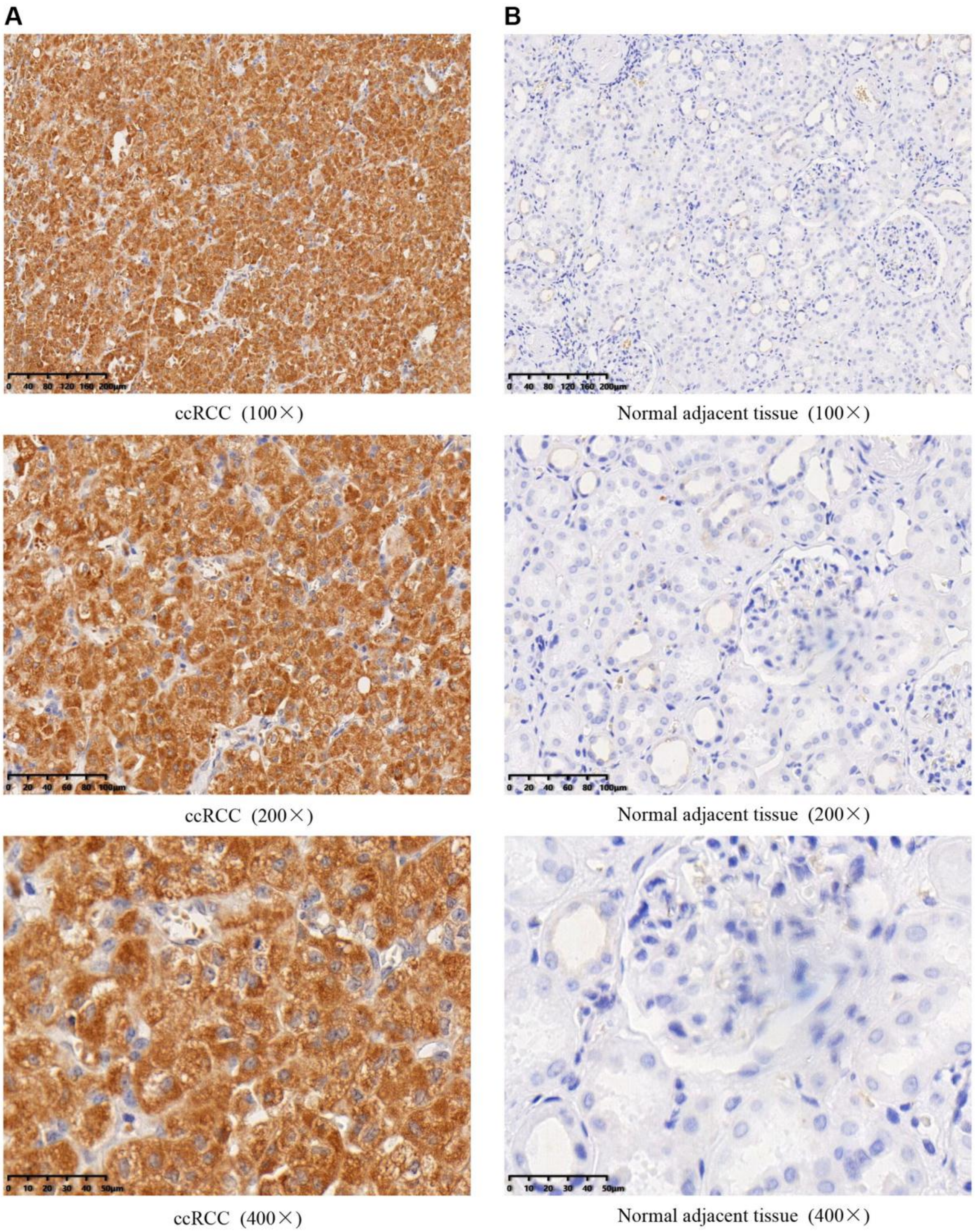

Supplementary Figure 1. The STK4 expression in ccRCC and normal adjacent tissue by IHC. The expression of STK4 in ccRCC (**A**) and normal adjacent tissue (**B**) in 3 different power representative immunohistochemical images (100×, 200×, and 400×).
